# Universal newborn hearing screening with automated auditory brainstem response (AABR) in Hungary: 5-year experience in diagnostics and influence on the early intervention

**DOI:** 10.1007/s00405-022-07441-4

**Published:** 2022-06-29

**Authors:** Anita Gáborján, Gábor Katona, Miklós Szabó, Béla Muzsik, Marianna Küstel, Mihály Horváth, László Tamás

**Affiliations:** 1 Department of Otorhinolaryngology, Head- and Neck Surgery, Semmelweis University, Szigony Street 36, 1083 Budapest, Hungary; 2 Department of Otorhinolaryngology, Heim Pál National Pediatric Institute, Budapest, Hungary; 3 1st Department of Pediatrics, Semmelweis University, Budapest, Hungary; 4 National Directorate General for Hospitals, Budapest, Hungary; 5 Doctoral School of Semmelweis University, Budapest, Hungary

**Keywords:** Neonatal hearing screening, Auditory brainstem response, AABR, Congenital hearing loss, Cochlear implant, National newborn hearing screening registry

## Abstract

**Purpose:**

In 2015 a new regulation and guidelines for the universal newborn hearing screening by AABR measurement have been implemented in Hungary. The aim of our study was to analyse (1) the past 5 years of data from our diagnostic centre about the incidence and types of congenital hearing losses, and (2) the first experiences with the National Newborn Hearing Screening Registry, started in 2019, and (3) the influence of the screening on the pediatric cochlear implant program.

**Methods:**

1269 children referred to our diagnostic centre between 2017 and 2021 were investigated. A third AABR measurement and full audiological evaluation were performed. Furthermore, one-year period data of the screening registry, and the number of implanted children at or under the age of 3 were analysed using the national databases.

**Results:**

Altogether 276 newborns (22% of the referred cases after the two-stage screening) had hearing loss, 134 (49%) out of them was conductive origin, almost twice frequent in male as in female. Permanent sensorineural hearing impairment was found in 142 (51%), 58 (40%) of them had bilateral, severe to profound hearing loss, occurring more frequently in male as in female. The national digital registration of the screening data within 12 months concerned 68%. The number of early cochlear implantation in one year increased from 1 to 23 children in the past 15 years.

**Conclusion:**

A third AABR after the two-stage screening increased the efficiency and filtered the 78% false-positive cases. The audiological diagnostics verified and typed the hearing losses ensuring the early intervention.

## Introduction

The early recognition of neonatal hearing impairment is very important. The development of the hearing system begins in a very early phase of the fetal life and even the intrauterin hearing initiates the sensation [1, 2]. The central auditory processing develops rapidly in the first 3 years of life, but after this period the decreasing plasticity of the brain gives less and less possibility to reach normal maturity of perception [3]. Normal hearing is necessary for speech understanding and language development outcomes. Hearing impairment without recognition and intervention causes an irreversible handicap, with delays in speech and language skills and disorders in social and emotional development [4, 5]. As the first months and years are essential for adequate linguistic outcomes, early recognition of hearing impairment is very important [6, 7].

The newborn hearing screening can ensure the discovery the congenital hearing losses [8–10]. The screening should be universal, therefore after several years of partially focused screening [11] we achieved a regulation of universal newborn hearing screening and we created the guidelines as well in 2015 in Hungary [12]. One of our goals was to introduce the national guidance and protocol for hearing screening, diagnosis and intervention. According to this programme, all the well-babies and the babies with risk factors are measured with AABR.

The aim of this study was to analyse the types and severity of the hearing losses diagnosed in babies referred to an audiological centre over a 5-year period. The patients were followed up regarding the intervention as well. It is known, that children who receive appropriate intervention, hearing aid under 6 months of age have a much better outcome in speech development, speech understanding, and even at school they have larger vocabularies. [4, 5]. According to our guidelines after the two-stage screening the babies with the failed result are referred to audiological centres, where all requirements for the early intervention are available.

The appropriate data collection would help to give precise information about the epidemiology of the congenital hearing loss, the judgement of the screening coverage, the calculation of the devices (hearing aid, cochlear implant, BAHA) and special education required for the rehabilitation and the cost-effectiveness of the screening and intervention. Therefore, we devised a national data collection, with a registry, that was improved in Hungary by informatics development. The National Newborn Hearing Screening Registry is able to collect and summarize the screening results from the hospitals, and the diagnostic results from the verification centres. According to these data, all newborn babies can be followed up and with a feedback system all undiagnosed cases can be attended. Our goal is to provide full access to newborn hearing screening and ensure for all children the early recognition of hearing loss and give early intervention.

Cochlear implantation is a chance for patients with profound hearing loss or deafness to create normal hearing [13]. Cochlear implant (CI) programme started in 1985 in Hungary [14]. A proper diagnosis could help children to get the necessary care, e.g. cochlear implant in early age. The good impact of screening could be seen in elevated numbers of CI surgeries in very young children.

## Methods

### Two-stage AABR screening

**Fig. 1:**
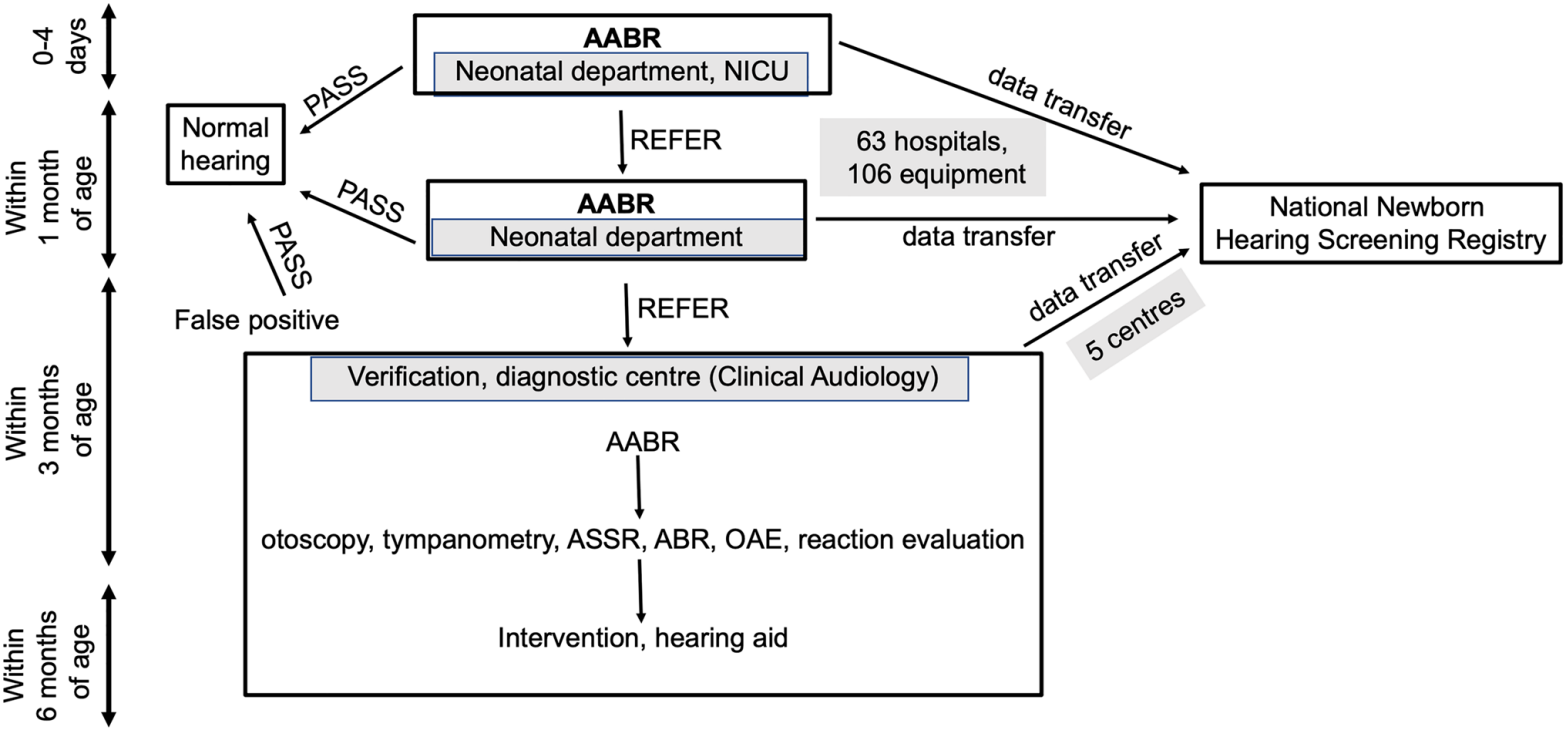
The algorithm of the newborn hearing screening and diagnostic and assessment phase. National screening protocol and diagnostic protocol of our audiological centre (see Footnote 1)

In the neonatal departments and neonatal intensive care units of the hospitals on the day 0–4 all the babies (normal and high-risk) are tested with AABR. The acoustic stimulation is a 35 dB CE-Chirp stimulus, with both ears tested. Children who do not pass the first screening are measured within the first month of age in the same hospital at an outpatient neonatal department [12]. The children with unilateral or bilateral failed results are referred to the audiological centres for verification and full audiological investigation and attendance (Fig. 1).

### Audiological assessment

The referred babies are guided to regional verification centres. In our clinical audiology^1^ after a third AABR measurement, physical investigation, tympanometry with high-frequency test tone (1000 Hz), in cases with normal pressure in the middle ear the objective hearing threshold is measured with the auditory steady-state response (ASSR) at octave frequencies from 500 to 4000 Hz simultaneously on both ears, clinical-ABR, otoacoustic emission (OAE), reaction evaluation subjective tests with our special teacher are performed (Fig. 1). In cases of B type tympanogram and a diagnosis of otitis media with effusion, the further diagnostic steps are performed after the healing. In cases of ear canal atresia and other cases with anomalies suspect on conductive hearing loss (e.g. ossification disorders, craniofacial malformations) or suspicion on syndromes mostly accompanying conductive hearing loss, bone conduction ASSR and ABR are performed as well.

The objective measurements are done in the natural sleeping of the patient in the first months of life. No anesthesia is used. This needs strong cooperation of the parents and sometimes a long waiting time for the medical staff. In cases of hearing loss further investigations are performed to explore the causes: genetic testing for connexin 26 with DNA sequence analysis of GJB2, imaging with CT, MRI, serology and searching for potential syndromes.

### Data collection and analysis

The audiological results of a 5-year period, 01 January 2017 to 31 December 2021 were analysed using the clinical database. According to our audiological investigations, the types and severity of the hearing losses were characterized. In cases of outer ear malformation with ear canal atresia permanent conductive hearing loss (atresia on charts) was diagnosed. Based on the physical investigation and with a B-type tympanogram the diagnosis was the conductive hearing loss caused by otitis media with effusion (OME). Without any pathological finding in the conductive system, with A-type tympanogram the diagnosis was sensorineural hearing loss (SNHL) and the average estimated hearing threshold regarding the objective threshold levels of 500, 1000, 2000, 4000 Hz was calculated. Mild hearing loss is diagnosed between 35 and 40 dB, moderate hearing loss 40–60 dB, severe between 60 and 80 dB and profound with more than 80 dB, respectively. The incidence of congenital hearing loss in male and female were compared.

The National Newborn Hearing Screening Register started in 2019. From the neonatal departments and NICUs there is an equipment-to equipment data transfer, supplied by different types of AABR informatically connected to the register with a special informatics solution. Analysing one-year (September 2020–August 2021) data according to the register we could calculate the efficacy of data collection. We compared the registered number of measurements with the number of births in Hungary in the same period.

As an assessment of early intervention, the number of children implanted at or under the age of 3 is presented according to the data of the National Health Insurance Fund.

### Audiological intervention

Based on audiological diagnosis the OME cases were followed up, children with ear canal atresia were given bone conductive hearing aid, the unilateral sensorineural hearing loss cases were observed, and in profound cases cochlear implantation was offered. In cases of bilateral sensorineural hearing loss depending on the severity hearing aid was given. Mild cases mostly do not need intervention, these cases were followed up, biannually they have audiological investigations. For moderate to profound hearing losses hearing aids were given. In cases of profound hearing loss—after providing a high-power hearing aid for some months—CI is offered.

## Results

In our clinic, in the past 5 years, 1269 children referred from newborn hearing screening were investigated. According to the AABR—as a third stage measurement—22% of the cases has been verified as hearing impairment, which means that 276 newborns had congenital hearing loss, the other 78% proved to be false-positive result after the two-stage screening at the hospitals. The verified hearing loss ratio increased year by year, from 15.9 to 26.7%.

According to the physical investigation and tympanometry of 134 newborn babies, 49% of the positive cases had conductive hearing loss. With B-type tympanogram of 121 children, 44% had a transient problem, caused by otitis media with effusion. Counting the incidence within our cases 80 boys, 66% of the OME cases and 41 girls, 34% of the cases were found (Table 1). Permanent conductive hearing loss, caused by ear canal atresia was diagnosed in 13 children, 5% of hearing losses, and the male/female ratio was 62%/38%.

**Table 1:** The incidence of hearing loss annually between 2017 and 2021 diagnosed in the clinical centre (see Footnote 1), and the summarized numbers of cases over the 5-years period. In the boxes, the total number and the number of male (M) and female (F) cases can be found

Type of hearing loss	2017	2018	2019	2020	2021	5 years
Conductive HL atresia	2 (M:0, F:2)	2 (M:2, F:0)	1 (M:0, F:1)	3 (M.1, F:2)	5 (M:5, F:0)	13 (M:8, F:5)
Conductive HL OME	32 (M:20, F:12)	27 (M:18, F:9)	18 (M:9, F:9)	20 (M:12, F:8)	24 (M:21, F:3)	121 (M:80, F:41)
SNHL unilateral	6 (M:3, F:3)	2 (M:1, F:1)	6 (M:1, F:5)	9 (M:4, F:5)	17 (M:9, F:8)	40 (M:18, F:22)
SNHL bilateral mild	1 (M:1, F:0)	4 (M:1, F:3)	1 (M:0, F.1)	4 (M:2, F:2)	2 (M:2, F:0)	12 (M:6, F:6)
SNHL bilateral moderate	6 (M:4, F:2)	7 (M:5, F:2)	3 (M:1, F:2)	8 (M:4, F:4)	8 (M:3, F:5)	32 (M:17, F:15)
SNHL bilateral severe	0	3 (M:1, F:2)	1 (M:1, F:0)	3 (M:3, F:0)	2 (M:1,F:1)	9 (M:6, F:3)
SNHL bilateral profound	15 (M:12, F:3)	11 (M:8, F:3)	6 (M:4, F:2)	9 (M:5, F:4)	8 (M:3, F:5)	49 (M:32, F:17)

**Fig. 2:**
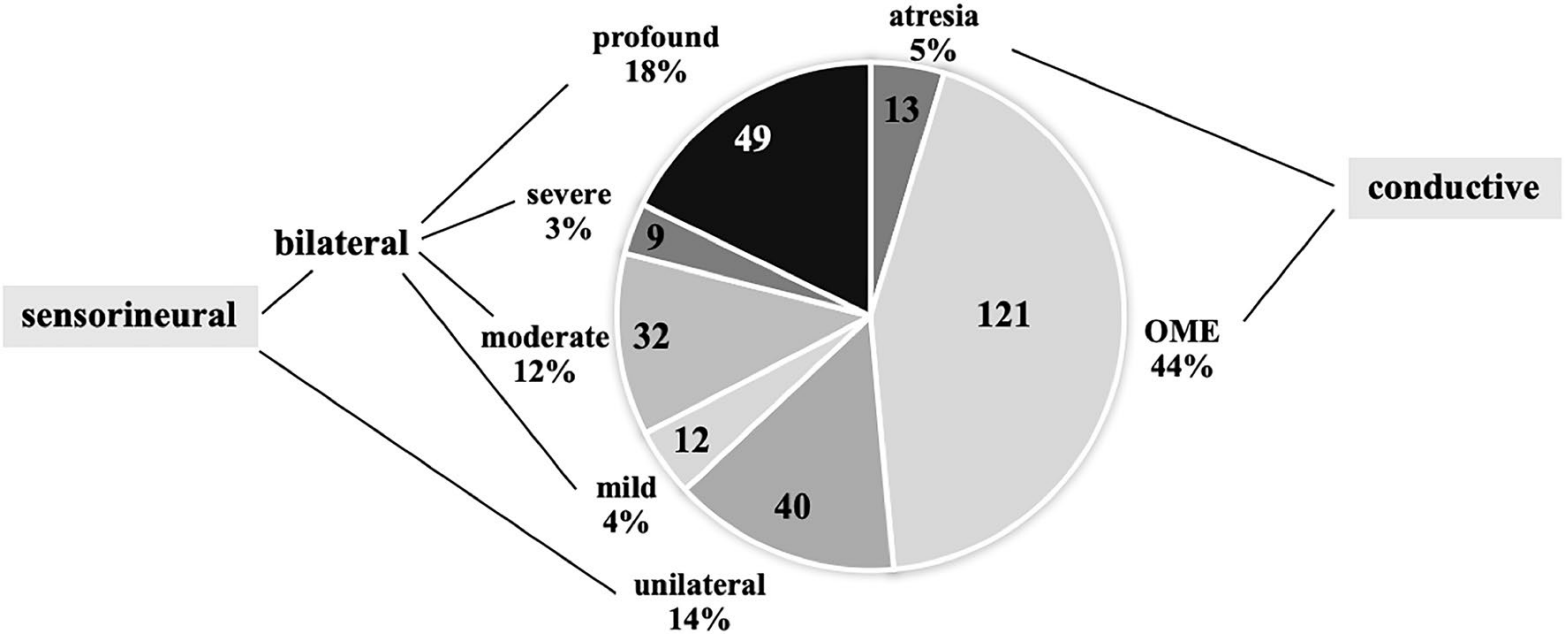
The proportion of different types and severity of the diagnosed hearing losses and numbers of children between 2017 and 2021, within 5 years in our centre (see Footnote 1)

Sensorineural hearing losses were diagnosed in 142 infants, that was 51% of the verified cases, 28% unilateral and 72% bilateral impairment were among them. (Fig. 2.) Regarding the severity of the bilateral hearing losses (from 102 children), in 12% (12 children) mild, in 31% (32 children) moderate, in 9% (9 children) severe and in 48% (49 children) profound hearing loss were diagnosed. The incidence of mild and moderate hearing losses was almost the same in males and females (23 boys and 21 girls), but the severe and profound hearing loss was much more frequent in males, as in females (38 boys and 20 girls). The diagnosis was established when they were 2–4 months old (Table 1.)

The National Newborn Hearing Screening Registry collects data from 63 hospitals in 49 cities all over the country, where the screening takes place with 106 pieces of AABR equipment at neonatal divisions and at intensive care units. (Fig. 1) In 1-year period the screening results of 61,039 newborns were registered, with their special identification number created for every child. In the same period, there were 89,876 births in these hospitals. That means that 68% of the expected data was transferred to the registry.

**Fig. 3:**
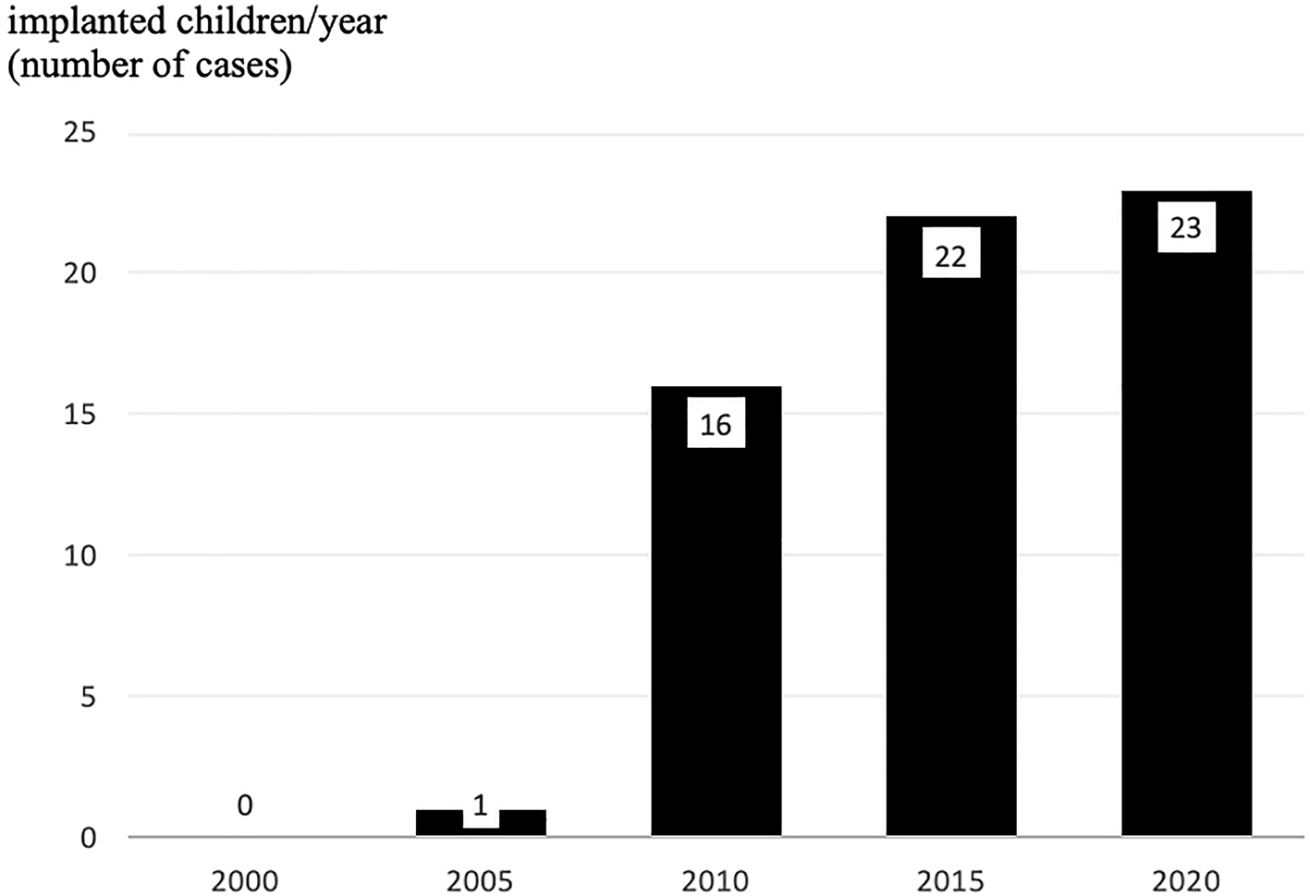
The numbers of cochlear implantation at age of ≤ 3 years in the years 2000, 2005, 2010, 2015 and 2020

The number of cochlear implantation in Hungary at or under the age of 3 years was only 1 case in 2005, and it has been growing to 23 cases in 2020. (Fig. 3.) We collected the present status of the 49 children diagnosed in our centre (see Footnote 1) within the 5-year period with bilateral profound hearing loss, among them 21 children were already implanted, 4 children are in the implant programme in progress. In ten cases the multidisciplinary diagnosis showed central origin (e.g. nervus vestibulocochlearis aplasia, hypoxic-ischaemic encephalopathy, and two children have died out of them), in two cases other illnesses (e.g. syndrome with severe heart disease, feeding difficulties) delay the safe operation. In eight cases the parents deny or refuse CI (e.g. deaf parents). The other four families have not returned to our clinic. However, in Hungary even the single-sided impairment could be implanted covered by the national insurance, in these cases the parents mostly do not want the cochlear implant for their children. At our clinic, only one child was implanted with single-sided deafness, but she had a progressive mild-moderate hearing loss on the other side. Bone conduction hearing aids (BAHA, Bonebridge) or CROS can be the other solutions, these rehabilitation options are discussed with the parents.

## Discussion

The early recognition and intervention of neonatal hearing impairment give the chance for all affected children for normal speech and language development. Different screening protocols exist for detecting congenital hearing loss. These protocols vary in the measuring methods, in the number and timing of measurements [15–18]. After decades of using subjectively judged behavioural hearing tests and only sporadic screening with objective method [11, 19], from 2015 screening with AABR is universally obligatory in Hungary. To ensure a high attendance rate the screening is hospital-based, performed by nurses at the maternity or NICU ward [12]. Special training programmes are organized for the nurses to improve their skills and awareness in hearing screening. Our national programme is a two-stage screening with AABR, one within the first 4 days after birth in the hospital, and in referred cases the second step is a rescreening at the neonatal department as an outpatient. The referral criteria is no response with AABR at 35 dB nHL at any side, therefore all types of hearing impairment, even the mild hearing loss cases and unilateral problems are recognized. Before creating the guidelines for national newborn hearing screening and deciding the method, the literature data were analyzed and preliminary data were collected. AABR was preferred to avoid false-negative diagnosis of auditory neuropathy and central origin hearing losses [17, 19, 20]. In the investigated 5 years at least 16 cases with central origin hearing loss were diagnosed according to the anamnestic data, but no simultaneous OAE and AABR were measured immediately after birth. The other aspect of choosing AABR was the lower false positive rate than with OAE-based screening at the neonatal departments, as it was described in the literature. [15, 21–24]. Our previous experience was the same with OAE screening, as the referral rate was 16% comparing the AABR referral rate, that became 1–2% after some months of experiences in a clinical neonatal department.

The newborns who failed at the screening are sent to one of the five verification centres. (Fig. 1) Analysing the results of the past 5 years in our centre (see Footnoe 1), 276 children were diagnosed with hearing loss from the 1269 referred cases. The third AABR performed by the audiological assistant at the clinical centre was judged to be effective in filtering the rest of the false-positive cases. This rate (the average for 5 years 78% false positive) is similar to other centres after the two-stage AABR protocol [25–28]. The rate is an indicator of the effectiveness of the 2-step screening. Regarding the data, year by year a decrease (from 84 to 73%) in false-positive rate could be observed. That means an increase in the effectiveness of the screening procedures. The impact of trainings for the nurses, the progress of awareness in this field, the improving function of national data collection and gained experiences could be behind this improvement. We could observe this augmentation, despite in the last two years the Covid-19 pandemic could have a negative impact on the newborn hearing screening [29]. Increasing the effectiveness, less children needs more time to wait and undergo full audiological assessment. After the third AABR, all the referred children had verified hearing loss. This third stage screening by professionals prevented the referral of 993 children for the clinical tests within 5 years. According to our regional follow-up study regarding the diagnostic results of these 5 years we can give epidemiological data about the occurrence of different types of congenital hearing impairment. Almost half of the hearing loss cases were diagnosed as transient conductive hearing loss caused by otitis media with effusion, which percentage is very close to the literature data [30–32]. The incidence of ear canal atresia (5%) is similar to other diagnostic centres [32]. Among the sensorineural hearing loss cases about one third had a one-sided problem with normal hearing on the other side. From the bilateral cases, more than half (57%) was severe or profound hearing loss. Regarding the ethiological factors, previously in a five-and-a-half years period the congenital profound hearing loss in 89 children who underwent cochlear implantation were investigated. The origin of the hearing loss was discovered in 62.9% of our patients, 38.8% out of them were allele frequency of c.35delG mutation [33]. 2018 regarding all the sensorineural hearing loss cases (unilateral, bilateral, mild to profound) the etiology in 52.9% of the cases could be discovered. 35.2% of the cases were connected to the pathogenic mutations of the GJB2 gene, the other reasons were meningitis, cytomegalovirus or other intrauterine infections, premature, hypoxia, ototoxic medication, neurodegenerative disorders, hyperbilirubinaemia, long NICU stay. The pathomechanism of 78% of all cases could be described, including conductive hearing loss cases with otitis media or ear canal atresia [34].

According to our guidelines, the screening should be performed up to 1 month of age, then we should give a diagnosis up to 3 months of age and we begin the rehabilitation, for example we give hearing aid up to 6 months of age for the children, if it’s possible. Children with bilateral hearing loss of moderate, severe or profound severity were supplied with hearing aid at their 4–10 months of age. The mild cases mostly do not need any correction in this early phase [35], but the strict follow-up is crucial to recognize a possible progression or later appropriate intervention. In case of profound hearing losses, after about half a year of using a hearing aid without efficiency, most of the children got into the cochlear implant programme or were implanted already. Regarding the pediatric cochlear implant programme the increase in the number of early implantation could be detected from year to year. This tendency is realised even though 2020 was a pandemic year when elective operations were cancelled for some months. These data show the efficacy and influence of the newborn hearing screening programme and the wider knowledge about the relevance of early intervention. The significant increase is partially due to the effective newborn hearing screening and the awareness in this field of health. The optimal timing for cochlear implantation is around 12 months of age, the goal of the programme is to ensure for children this early implantation when it’s needed. Sometimes other diseases delay the hearing diagnostics and intervention. We try to minimize the developmental delay, as the earlier the intervention the better the outcome in the field of speech development and even the school, language, social, behavioural development. With our protocol, even mild and moderate hearing losses are established and their early aiding and follow up are clearly beneficial for these children [5, 35].

Unexpectedly, we found a significant gender difference in the incidence of congenital hearing impairments. Several studies have now shown equal AOM prevalence in males and females [22] and many previous studies had shown higher incidence in boys [36–38]. In our study almost twice was the numbers of male (66% of the cases) than in female (34% of the cases) with conductive hearing loss caused by OME. Among the children with bilateral severe and profound sensorineural hearing loss much more boys (66% of the cases) than girls (34% of the cases) were found as well.

With starting the National Newborn Hearing Screening Register the goal was to find all children who need help due to hearing impairment. During the international data collection by the EUSCREEN project [39, 40], a lot of investigated data were unavailable, but with continuously improving the efficacy of national data collection started in 2019 we can calculate more and more results. Innovative solutions were found to harmonize the measurement results coming from different types of AABR device, and through these informatic channels now we have an equipment-to-equipment connection. Our experience shows that an innovation in the screening equipment could be recommended in extending the functions in data transfer to registers. Our national registering coverage of 68% is satisfying after the experience of one year but should be further improved to avoid the loss of failed children and miss the early intervention. The data uploading from the screening machines to the hospital information system is semi-manual (wired network needed). We train the staff, but unfortunately, the pandemic set back this process. There is a learning curve for NICU and neonatal department nurses using the screening system, and reporting. As the courses and trainings are restarting the report-rate will be improved surely by time and practice. With appropriate data collection, we could calculate the number of hearing aid and cochlear implant, and other implanted hearing aids (BAHA), speech and language therapists needed annually for children. These data are very important to provide national statistics of incidence as well. We can provide information about the screening sensitivity, specificity, cost-effectiveness and epidemiological data. An international, standardized register would help the comparison [39–41].

Although we have the guidelines for newborn hearing screening, the appropriate amount of equipment, educated health professionals, national data collection, we can always, even nowadays, find late-diagnosed children, who could not get appropriate interventions at the time that could have improved their language development. The cause of these “late” cases can be delayed diagnosis or delayed intervention. Behind these delays could stand lack, not enough or not appropriate information, fear, worry, concern, denial, lack of awareness, low education of the parents or bad social circumstances. In some cases, children are not sent to the centres, in other cases the parents do not want the appropriate intervention, for example, the cochlear implant. We face these problems however we have the universal newborn hearing screening and financial support, as the intervention is totally financed by the government health insurance. What we need is a more accurate follow-up system and we should give more information, even in the healthcare system to the specialists and even to the public, for example in the social media and education. The parents’ and professionals’ awareness of the possibility of full rehabilitation and their knowledge about the consequences of the delayed diagnosis have to be enhanced.

## Abbreviations

AABR: Automated auditory brainstem response
ASSR: Auditory steady state response
OAE: Otoacoustic emission
OME: Otitis media with effusion
NICU: Neonatal intensive care unit
CI: Cochlear implant
BAHA: Bone anchored hearing aid

## Footnotes

1 In our clinical audiology and in our centre and in the clinical centre means the Audiology of the Department of Otorhinolaryngology, Head and Neck Surgery at Semmelweis University (removed for blind peer review).
